# Associations Between Altered Auditory EEG Markers and Clinical Impairments in Fragile X Syndrome

**DOI:** 10.1007/s10803-025-07076-4

**Published:** 2025-10-06

**Authors:** Mélodie Proteau-Lemieux, Inga S. Knoth, Saeideh Davoudi, Charles-Olivier Martin, Anne-Marie Bélanger, Valérie K. Fontaine, Hazel Maridith Barlahan Biag, Leonard J. Abbeduto, Sébastien Jacquemont, David Hessl, Randi J. Hagerman, Andrea Schneider, François V. Bolduc, Sarah Lippé

**Affiliations:** 1 Department of Psychology, University of Montreal, Montreal, QC, Canada; 2 Azrieli Research Center of the Sainte-Justine University Hospital, Montreal, QC, Canada; 3 Department of Neuroscience, University of Montreal, Montreal, QC, Canada; 4 Department of Pediatrics and MIND Institute, University of California Davis School of Medicine, Sacramento, CA, USA; 5 Department of Psychiatry and Behavioral Sciences and MIND Institute, University of California Davis School of Medicine, Sacramento, CA, USA; 6 Department of Pediatrics, University of Montreal, Montreal, QC, Canada; 7 Department of Pediatrics, University of Alberta, Edmonton, AB, Canada

**Keywords:** Fragile X syndrome, EEG, AEPs, Neurodevelopment, Cognition, Behavior

## Abstract

**Purpose:**

Individuals with Fragile X syndrome (FXS) manifest clinical impairments in several domains. Previous research has shown that auditory evoked potentials (AEPs), measured using electroencephalogram (EEG), are altered in FXS, but the associations between these alterations and the symptoms observed in FXS have not been thoroughly investigated. The aim of this study was to compare AEP markers between individuals with FXS and neurotypical (NT) controls, with the main purpose of exploring how these markers are related to various clinical symptoms present in FXS.

**Methods:**

A passive auditory oddball paradigm was presented. P1, N1, P2, N2, P3 and mismatch negativity (MMN) amplitudes and latencies were compared between 41 children and adults with FXS and 46 age-matched NT controls. Amplitudes and latencies, as well as habituation and change detection effects were compared between the groups using mixed design ANOVAs. Pearson correlations were then performed to explore associations between AEP markers and symptoms in the FXS group.

**Results:**

Our results showed that FXS participants had increased N1, P2 and MMN amplitudes and latencies, as well as lack of habituation and change detection effects compared to NT controls. Our correlational analyses revealed several associations between AEPs and phenotypic manifestations; notably, associations between exaggerated N1 and P2 amplitudes and more severe autistic and ADHD symptoms.

**Conclusions:**

These findings confirm that abnormalities of the N1 and P2 components are robust biomarkers of altered sensory processing in FXS and suggest that these alterations may present a dose–response relation to clinical impairments in FXS.

Fragile X syndrome (FXS) is an X-linked genetic condition caused by a mutation of the *Fragile X Messenger Ribonucleoprotein gene 1* (*FMR1*) on the X chromosome, consequently leading to the loss of its expressed protein, the *FMR1* protein (FMRP). Females with FXS are generally not as severely impacted because they carry an unaffected X chromosome. FXS is the leading hereditary cause of intellectual disability (ID) and the most frequent monogenic cause of syndromic autism spectrum disorder (ASD) (Hagerman et al., 2012). Around 85% of males and 25–30% of females with FXS meet the criteria for ID (Chonchaiya et al., 2009; Lozano et al., 2014), which is characterized by an intellectual quotient (IQ) below 70 and adaptive behavior challenges. Autistic symptoms are also very prevalent in FXS. Around 60% of males with FXS have a cooccurring diagnosis of ASD, while 90% manifest autistic-like behavior (Hernandez et al., 2009; Marlborough et al., 2021), and approximately 20% of females with FXS meet the criteria for ASD (Marlborough et al., 2021). The main ASD symptoms observed in FXS are delayed communication abilities, repetitive behaviors, as well as impaired social functioning. The presence of ASD traits in FXS is associated with more severe developmental delays (Richards et al., 2015). Moreover, around 80% of males and 30% of females with FXS have symptoms of attention deficit hyperactivity disorder (ADHD) and manifest severe attentional deficits (Lozano et al., 2014). Lastly, aberrant behavior, including irritability and impulsivity, as well as symptoms of depression and anxiety, are also very common in FXS (Chonchaiya et al., 2009; Schneider et al., 2009).

In addition, individuals with FXS manifest neurophysiological alterations. One of the hallmarks of FXS is hyperexcitability, which is thought to be at the root of many neurological and psychiatric impairments observed in individuals with FXS (Contractor et al., 2015). Specifically, hyperexcitability is caused by a reduced activation of the GABAergic system, thus leading to excessive neuronal excitability (Gao et al., 2018). Moreover, hyperexcitability of auditory processes is a robust biomarker in animal models of FXS (Rotschafer & Razak, 2014), and auditory hypersensitivity, a core symptom of FXS, is thought to be directly related to cortical hyperexcitability (Razak et al., 2021). Studies investigating electrocortical abnormalities with transcranial magnetic stimulation (TMS) showed exaggerated responses and reduced intracortical inhibition in FXS (Morin-Parent et al., 2019; Proteau-Lemieux et al., 2021), while electrodermal studies showed lack of habituation to sensory stimuli in FXS (Miller et al., 1999). Electroencephalogram (EEG) studies investigating auditory and visual event-related potentials (ERPs) showed severe alterations in both modalities, suggesting that cortical hyperexcitability could be at the source of deficient sensory processing in individuals with FXS (Côté et al., 2021; Ethridge et al., 2016; Knoth et al., 2014, 2018; Schneider et al., 2013; Van der Molen et al., 2012a, 2012b).

Previous EEG studies using passive oddball paradigms, which consist in the presentation of repeated stimuli followed by a deviant, reported altered auditory evoked potentials (AEPs) of the N1, P2, and N2 components. Specifically, increased amplitudes and latencies were observed in children and adults with FXS (Castrèn et al., 2003; Ethridge et al., 2016, 2020; Knoth et al., 2014, 2018; Sinclair et al., 2017; St. Clair et al., 1987; Van der Molen et al., 2012a, 2012b), as well as increased N1/P2 peak-to-peak amplitudes (Côté et al., 2021). Moreover, habituation, a process implicated in learning mechanisms and characterized by a decrease in brain activity in response to the presentation of a repeated stimulus, was also found to be altered in FXS. A decrease in N1 and N2 habituation was mostly found in FXS participants compared to neurotypical (NT) controls (Castrèn et al., 2003; Ethridge et al., 2016; Sinclair et al., 2017; Van der Molen et al., 2012a, 2012b), although Ethridge and colleagues found a larger N1 habituation effect in FXS (2020). Another study observed N1 and P2 habituation in individuals with FXS and NT controls, but no differences between the groups (Ethridge et al., 2019). Côté and colleagues (2021) found N1/P2 and P2/N2 peak-to-peak habituation effects in both FXS and NT controls. Whereas the N1, P2, and N2 components have been extensively examined in FXS, the literature on P1 is scarce. One study investigated P1 amplitudes and habituation between FXS and NT children. This study found no difference in P1 amplitudes between groups in the frontal region, but in the temporal region, FXS children showed higher P1 amplitudes (An et al., 2022). P1 habituation was not found in either group. Another study found no differences in P1 amplitudes between FXS individuals and NT controls (Ethridge et al., 2020). Furthermore, change detection, a sensory process reflected by higher amplitudes following the presentation of a deviant stimulus, was shown to be reduced in males with FXS (Van der Molen et al., 2012a, 2012b). The P3 component is an important change detection measure characterized by a heightened neural response observed after the presentation of a deviant tone and is thought to involve attentional and working memory processes (Devitt et al., 2015). The earlier P3 subcomponent, P3a, is elicited by an infrequent tone and reflects the involuntary shift in attention, while the later subcomponent, P3b, also occurs after an infrequent tone, but is elicited by a voluntary attentional shift (Friedman et al., 2001; Squires et al., 1975). In FXS, P3a results are inconsistent. There is evidence of reduced P3 amplitudes following deviant tones in adults with FXS compared to NT controls (St. Clair et al., 1987). However, Van der Molen and colleagues () investigated the P3 subcomponents distinctively in adult males with FXS and NT adult males and found conflicting results. They found that P3a amplitudes where higher for deviant compared to standard stimuli in both males with FXS and NT males, indicating a change detection effect in both groups, while males with FXS showed decreased P3a amplitudes for the deviant stimuli, as well as longer latencies for both type of stimuli compared to NT males (Van der Molen et al., 2012a, 2012b). In their other study (Van der Molen et al., 2012a, 2012b), they found decreased P3b amplitudes in males with FXS for standard and deviant stimuli compared to NT males. Lastly, mismatch negativity (MMN) is another cognitive component reflecting change detection (Näätänen et al., 2014), and while literature on MMN in FXS is scarce, inconsistencies were observed in previous studies. MMN was found to be reduced in adult males with FXS in one study (Van der Molen et al., 2012a, 2012b), whereas another study found no differences in MMN amplitudes between individuals aged 4–51 years old with FXS and age-matched NT controls (Ethridge et al., 2020).

ERPs are the most commonly used features to investigate EEG abnormalities in individuals with FXS, and their translational potential is well recognized (Devitt et al., 2015; Kenny et al., 2022). Overall, previous EEG results using oddball paradigms indicate impairments in early sensory and attentional processes in individuals with FXS. Thus, exploring the associations between AEP alterations and the severity of core symptoms defining the behavioral phenotype in a cohort of individuals with FXS could help us better understand the cortical mechanisms underlying the clinical phenotypes present in this population. This study first aimed to investigate the N1, P1, P2, N2, P3a, and MMN components with a passive auditory oddball task in individuals with FXS and NT controls. We hypothesized that FXS individuals would show larger N1, P1, P2, N2 and P3a amplitudes and longer latencies for both standard and deviant stimuli, but reduced habituation and change detection effects compared to NT controls. Given the cognitive nature of MMN and that our FXS cohort manifested altered cognitive functioning, we also hypothesized that individuals with FXS would present reduced MMN amplitudes, as well as longer MMN latencies. The main objective was then to explore the associations between AEP abnormalities and the severity of symptoms present in FXS. We believe that individuals with FXS presenting increased alterations in auditory processing will also manifest more severe clinical symptoms.

## Methods

### Participants

Forty-one participants with a diagnosis of FXS aged between 7 and 34 years and 46 NT controls with a similar age distribution participated in the study. Table 1 provides the demographics of the study population. Genetic screening for FXS was performed prior to the study by a physician and a diagnosis was given when individuals presented > 200 repeats (full mutation) of cytosine-guanine-guanine (CGG). FXS participants were recruited via a multi-site, large-scale clinical study taking place at the CHU Sainte-Justine in Montréal, the University of Alberta, and the MIND Institute at the University of California, Davis. Inclusion criteria to participate in the trial included a non-verbal intelligence quotient (NVIQ) < 85, as measured with the Leiter-III. Data from NT controls were collected via previous studies that took place at the NED laboratory at CHU Sainte-Justine.

### Neuropsychological Measures in the FXS Cohort

The Leiter-III (Roid et al., 2013) was used to assess the NVIQ of FXS participants. The Autism Diagnostic Observation Scale-Second Edition (ADOS-2) (Lord et al., 2012) was administered to quantify autistic traits and determine the presence of cooccurring ASD in our participants. The ADOS-2 comprises four different modules and the choice of module is based on chronological age and language abilities. Modules 1, 2, and 3 assess social affect, as well as restricted and repetitive behaviors, while the 4th module evaluates communication and social interaction. To allow comparability between the different modules, the revised algorithm for Module 4 was used (Hus & Lord, 2014). Adaptive behavior was evaluated with the Vineland Adaptive Behavior Scales-Third Edition (VABS-3) (Sparrow et al., 2016), while behavioral impairments were evaluated with the Fragile X scoring algorithm of the Aberrant Behavior Checklist for Community (ABC-C-FX) (Sansone et al., 2012). The Anxiety Depression and Mood Scale (ADAMS) (Esbensen et al., 2003) was used to screen for anxious and depressive symptoms. Lastly, the Swanson, Nolan, and Pelham Questionnaire (SNAP-IV) is based on DSM-V criteria for ADHD (Hall et al., 2020) and was used to identify participants with ADHD symptoms. For the Leiter-III and VABS-3, higher scores are associated with better functioning, whereas higher scores on the other instruments are associated with more severe problems. Table 2 provides the co-occurring diagnoses for ID, ASD, and ADHD in the clinical population. Table 3 indicates the pharmacological treatments taken by the participants with FXS. Supplementary File 1 provides the mean scores for the neuropsychological instruments, and the domains/subscales/dimensions assessed by each instrument in the clinical population.

### EEG Procedure, Apparatus, and Stimuli

Recordings were carried out in EEG chambers at the three implicated sites. During net installation and recording, participants watched a silent movie to encourage compliance and help them stay calm. Two different EEG systems were used. An EGI 128-electrode dense array system (Magstim EGI, Eugene, OR, USA) was used for participants recorded at CHU Sainte-Justine and the University of Alberta, while a Brain Products 32-electrode ActiCAP (Brain Products, Germany) was used for participants tested at the MIND Institute. For the EGI system, Cz was used as online reference, while FCz was used as online reference for the Brain Products system. At each site, a sampling rate of 1000 Hz was used to record data, with a bandpass filter of 0.1–500 Hz. Impedances were kept below 40 kΩ and below 10 kΩ for the EGI and Brain Products systems, respectively. The passive auditory oddball paradigm consisted of 450 sinusoidal tones, where 390 ‘standard’ tones (1000 Hz; 86.67% of stimuli) and 60 ‘deviant’ tones (2000 Hz; 13.33% of stimuli) were presented at a sound intensity of 70 dB. Each tone was 70 ms in length, including a 10 ms rise/fall, and interstimulus and inter-trial intervals of 1000 ms. Ten different trials were presented, in which the deviant tone emerged pseudo-randomly at either the 5th, 6th, 7th, 8th, 9th, or 10th position, and each trial was presented 10 times at random. At the CHU Sainte-Justine and University of Alberta, auditory stimuli were presented using E-Prime 2.0 (Psychology Software Tools, Inc., Pittsburgh, PA, USA), while the Presentation software (Neurobehavioral Systems, Albany, CA) was used to present the auditory task at the MIND Institute.

### EEG Analyses

MATLAB software (version R2020b) with the EEGLAB toolbox (version 14.1.2) was used to carry out offline pre-processing analyses. Data was filtered using a 0.5–150 Hz high- and low-pass filter, and a 60 Hz notch filter was applied. For the EGI system, 28 external channels were removed because of the low-quality signal observed around the face, neck, and ears. Six corresponding external channels were removed for the Brain Products system. Electrodes with standard deviations < 2 μV, to remove electrodes with flat signal, and > 120 μV were automatically removed, and the remaining noisy electrodes were removed manually upon visual inspection. Data were then re-referenced to the average reference, independent component analysis (ICA) was carried out, and components presenting blinks, saccades and cardiac activity were manually removed. Data were segmented for each stimulus into 2000 ms epochs, with a − 1000 to 0 ms pre-stimulus onset. Epochs containing voltage amplitudes < − 200 μV and > 200 μV were automatically removed, and upon visual inspection, epochs with remaining artifacts were manually removed. The % of kept trials did not significantly differ between groups for any stimulus presentation (see Supp. File 2). The central (Cz) and fronto-central (FCz) regions were chosen as regions of interest (ROIs).

For AEPs detection, epochs were segmented into 800 ms trials, with a − 200 ms pre-stimulus baseline, and a 600 ms time window after stimulus onset. For the analyses, the 1st standard stimulus (S1) of each trial, the standard stimuli directly preceding a deviant tone (SPrecDev), and the deviant stimuli (Dev) were considered. A − 200 to 0 ms baseline correction was applied and trials were averaged for each presentation of the standard (S1 and SPrecDev) and deviant (Dev) stimuli. As a last step, and given that different EEG systems were used, data normalization was applied for each participant individually using z-scores (Davoudi et al., 2021). For P1, N1, P2, N2, and P3a components, specific time windows based on FXS literature (Van der Molen et al., 2012a, 2012b) and inspection of group averages were selected. Here, P1 was defined as the most positive peak between 50 and 150 ms, N1 was defined as the most negative peak between 80 and 150 ms, P2 was defined was the most positive peak between 150 and 250 ms, N2 was defined as the most negative peak between 200 and 400 ms, and P3a was defined as the most positive peak between 250 and 450 ms. P1, N1, P2 and N2 habituation were assessed between S1 and SPrecDev, while change detection was measured between SPrecDev and Dev for P1, N1, P2, N2, and P3a. The MMN component was calculated by first subtracting the average of the standard responses preceding the deviant stimuli from the average of the deviant response (Dev-SPrecDev). A difference curve was then created and MMN was defined as the most negative peak between 100 and 350 ms. Participants’ values were generated for each stimulus type (S1, SPrecDev and Dev), and peaks were computed semi-automatically within the time windows described previously. Lastly, components’ amplitudes and latencies were identified for each participant individually in the selected ROIs (Cz and FCz).

### Statistical Analyses

Statistical analyses were performed using SPSS Statistics version 29 (IMB Corp., Armonk, NY, USA). Skewedness and kurtosis criteria were used to determine normality of data distribution, and values between −1 and 1 were considered acceptable. For habituation, repeated measures ANOVAs with Habituation as the within-subject factor, Group as the between-subject factor, and Age as covariate were performed for amplitude and latency values. For change detection, mixed ANOVAs with Change detection as the within-subject factor, Group as the between-subject factor, and Age as covariate were carried out for amplitudes and latencies. Bonferroni corrections were applied for the 2X2 repeated measures and mixed ANOVAs to correct for multiple comparisons. For MMN amplitudes and latencies, 2 × 1 ANCOVAs with Age as covariate were performed, and Bonferroni corrections for multiple comparisons were used. Pearson correlations using a 5% False Discovery Rate (FDR) to correct for multiple correlations were carried out to explore the associations between AEPs and clinical impairments. Sex was added as a covariate in all correlational analyses while age was used as covariate for the correlations with instruments that do not include age-based norms (ABC-C-FX, ADAMS, and SNAP-IV). A 5% (*p* < .05) significance level was set for all analyses.

## Results

**Fig. 1:**
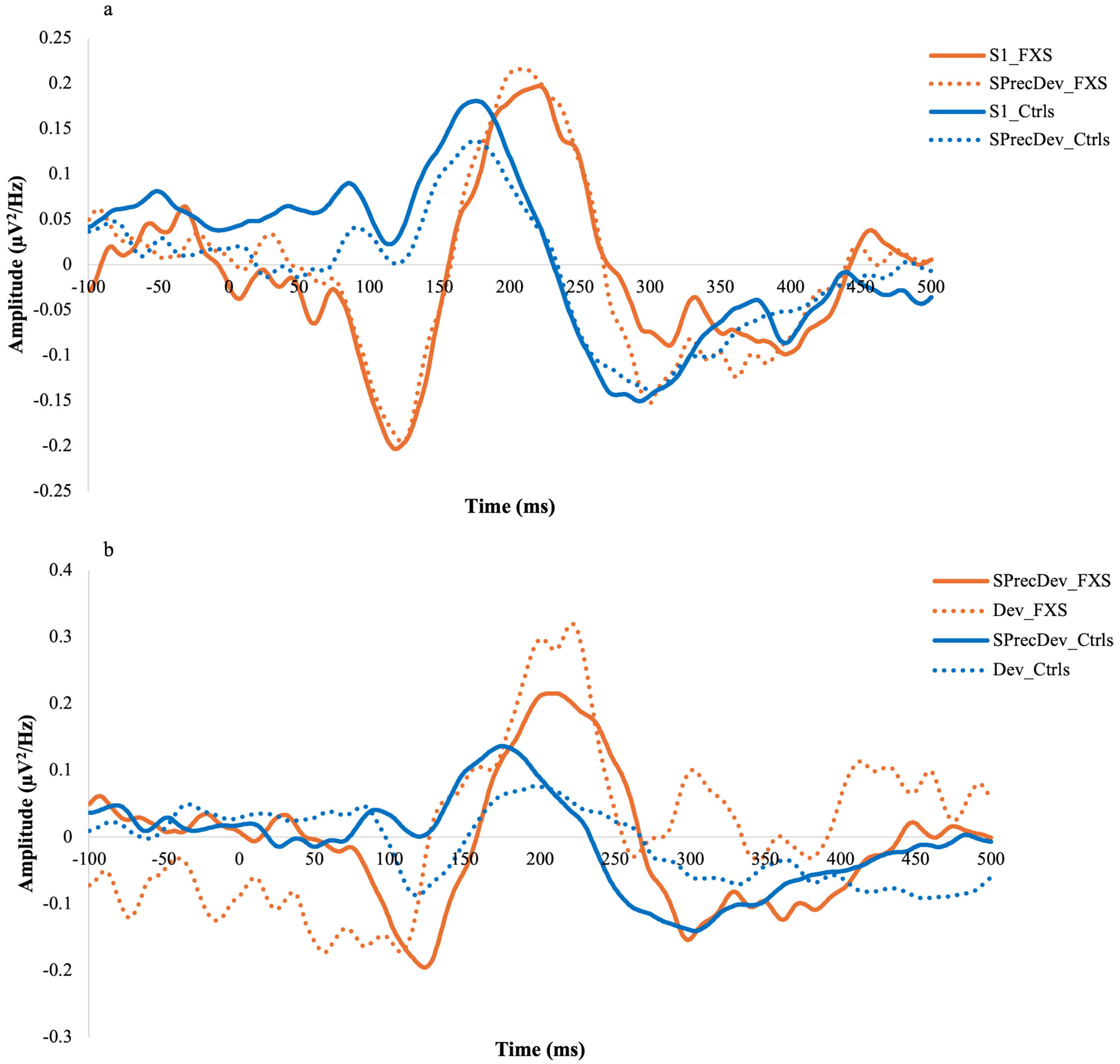
Grand average waveforms for S1 (bold lines) and SPrecDev (dotted lines) in FXS (orange) and NT controls (blue) in Cz (**a**) and grand average waveforms for SPrecDev (bold lines) and Dev (dotted lines) in FXS (orange) and NT controls (blue) in Cz (**b**)

Summaries of results for the AEP components between groups (Supp. File 3) and the correlations between AEPs and phenotypic manifestations in the FXS cohort (Supp. File 4) are presented in supplementary material. Figure 1 shows the grand average waveforms for S1 and SPrecDev for FXS and NT controls in Cz (a) and the grand average waveforms for SPrecDev and Dev for FXS and NT controls in Cz (b).

### Habituation (Response to Standard Stimuli)

#### P1

In Cz, a main effect of Age (*F*_(1,79)_ = 6.12, *p* = .015, *η*^2^ = .07) was found, indicating a decrease in amplitudes with age. A Habituation X Group interaction (*F*_(1,79)_ = 5.02, *p* = .028, *η*^2^ = .06) was also observed. S1 amplitude was lower in individuals with FXS (*p* = .01), and a P1 habituation effect was observed in NT controls only (*p* = .014). In FCz, a main effect of Age (*F*_(1,78)_ = 4.78, *p* = .032, *η*^2^ = .06) was found, where amplitudes decreased with age. Exploration of habituation in each group showed a P1 habituation in NT controls only (*p* = .036). Latency in Cz showed a main effect of Group (*F*_(1,84)_ = 11.38, *p* = .001, *η*^2^ = .12), indicating shorter latencies in individuals with FXS for SI (*p* = .013) and SPrecDev (*p* = .008). In FCz, a main effect of Group (*F*_(1,84)_ = 4.98, *p* = .028, *η*^2^ = .06) was observed, but pairwise comparisons showed no differences between groups.

#### N1

Results showed main effects of Age (*F*_(1,80)_ = 31.71, *p* < .001, *η*^2^ = .28) in Cz and FCz (*F*_(1,81)_ = 35.25, *p* < .001, *η*^2^ = .30), with amplitude decreasing with age in both ROIs. Main effects of Group were also found in Cz (*F*_(1,80)_ = 38.25, *p* < .001, *η*^2^ = .32) and FCz (*F*_(1,81)_ = 33.63, *p* < .001, *η*^2^ = .29). In both ROIs, amplitudes for S1 and SPrecDev were higher in individuals with FXS (all *p* < .001). N1 habituation was not found in either group. For latency, a main effect of Group was found in Cz (*F*_(1,84)_ = 13.85, *p* < .001, *η*^2^ = .14), indicating a longer S1 latency in individuals with FXS (*p* < .001). In FCz, a main effect of Group (*F*_(1,83)_ = 7.12, *p* = .009, *η*^2^ = .08) and a Latency X Group interaction (*F*_(1,83)_ = 7.00, *p* = .01, *η*^2^ = .08) were found. S1 latency was longer in individuals with FXS (*p* < .001), and latency tended to shorten from S1 to SPrecDev in individuals with FXS, although it was not significant (*p* = .057).

#### P2

A main effect of Group (*F*_(1,84)_ = 6.86, *p* = .01, *η*^2^ = .08) was observed in Cz, and amplitudes were higher in individuals with FXS for S1 (*p* = .032) and SPrecDev (*p* = .014). In FCz, a main effect of Age (*F*_(1,79)_ = 4.17, *p* = .044, *η*^2^ = .05) indicated increasing amplitudes with age. Also in FCz, a Group effect (*F*_(1,79)_ = 13.66, *p* < .001, *η*^2^ = .15) was observed. Higher amplitudes were found in individuals with FXS for S1 (*p* = .015) and SPrecDev (*p* < .001), and exploration of habituation in each group showed a small P2 habituation effect in NT controls only (*p* = .05). For latency, main effects of Group were observed in Cz (*F*_(1,83)_ = 16.77, *p* < .001, *η*^2^ = .17) and FCz (*F*_(1,84)_ = 22.52, *p* < .001, *η*^2^ = .21). In both Cz and FCz, individuals with FXS had longer S1 latencies (both ROIs *p* < .001), as well as longer SPrecDev latencies in Cz (*p* = .015) and FCz (*p* = .002).

#### N2

For amplitudes, only main effects of Age were found in Cz (*F*_(1,81)_ = 7.91, *p* = .006, *η*^2^ = .09) and FCz (*F*_(1,80)_ = 16.67, *p* < .001, *η*^2^ = .17), with amplitudes increasing with age. For latencies, only a main effect of Age was found in Cz (*F*_(1,84)_ = 10.71, *p* = .002, *η*^2^ = .11), where latencies lengthened with age. A main effect of Age (*F*_(1,84)_ = 9.86, *p* = .002, *η*^2^ = .11) was found in FCz, indicating increasing amplitudes with age. A Group effect (*F*_(1,84)_ = 4.51, *p* = .037, *η*^2^ = .05) was found in FCz. However, pairwise comparisons revealed no differences between groups.

### Change Detection (Response to Deviant Stimuli)

#### P1

For amplitudes, main Age effects were observed in Cz (*F*_(1,81)_ = 7.18, *p* = .009, *η*^2^ = .08) and FCz (*F*_(1,80)_ = 5.90, *p* = .017, *η*^2^ = .07), where amplitudes decreased with age. For latencies, a Group effect was observed in Cz (*F*_(1,84)_ = 5.56, *p* = .021, *η*^2^ = .06), and SPrecDev latency was shorter in individuals with FXS (*p* = .008).

#### N1

A main effect of Age (*F*_(1,81)_ = 19.51, *p* < .001, *η*^2^ = .19) was observed in Cz, as amplitudes decreased with age. A Group (*F*_(1,81)_ = 27.81, *p* < .001, *η*^2^ = .26) effect was observed in Cz, where amplitudes were higher in individuals with FXS for SPrecDev and Dev (both *p* < .001). In FCz, main effects of Age (*F*_(1,81)_ = 35.83, *p* < .001, *η*^2^ = .31), where amplitudes decreased with age, and Group (*F*_(1,81)_ = 15.55, *p* < .001, *η*^2^ = .16) were observed, indicating higher amplitudes in individuals with FXS for SPreDev (*p* < .001) and Dev (*p* = .01). No effects were found for latency.

#### P2

A main effect of Group was observed in Cz (*F*_(1,81)_ = 7.85, *p* = .006, *η*^2^ = .09). SPrecDev and Dev amplitudes were higher in individuals with FXS (both *p* = .02). A main effect of Group was also observed in FCz (*F*_(1,76)_ = 19.19, *p* < .001, *η*^2^ = .20), and amplitudes were higher in individuals with FXS for SPrecDev (*p* < .001) and Dev (*p* = .002). For latencies, a main effect of Age was observed in Cz (*F*_(1,84)_ = 7.66, *p* = .007, *η*^2^ = .08), where latencies lengthened with age. In FCz, main effects of Age (*F*_(1,84)_ = 11.81, *p* < .001, *η*^2^ = .12) and Group (*F*_(1,84)_ = 10.26, *p* = .002, *η*^2^ = .11) were observed. SPrecDev latency was longer in individuals with FXS (*p* = .002).

#### N2

In Cz, a main effect of Age (*F*_(1,81)_ = 6.06, *p* = .016, *η*^2^ = .07) was observed as amplitudes increased with age. Also in Cz, a Group effect (*F*_(1,81)_ = 5.20, *p* = .025, *η*^2^ = .025) was observed, where Dev amplitudes tended to be higher in individuals with FXS, but it did not reach significance (*p* = .06). In FCz, only a main effect of Age (*F*_(1,79)_ = 6.94, *p* = .01, *η*^2^ = .08) was found, where amplitudes increased with age. For latencies, a main effect of Age was found in Cz (*F*_(1,84)_ = 5.42, *p* = .022, *η*^2^ = .06), and main effects of Age (*F*_(1,84)_ = 5.00, *p* = .028, *η*^2^ = .06) and Group (*F*_(1,84)_ = 5.52, *p* = .021, *η*^2^ = .06) were observed in FCz, and latencies lengthened with age. However, no differences were found between groups.

#### P3a

In Cz, a main effect of Age (*F*_(1,73)_ = 5.83, *p* = .018, *η*^2^ = .07) was observed, where amplitudes increased with age. Pairwise comparisons indicated a difference in Change Detection (*p* = .018), and exploration of change detection in each group showed that amplitudes increased from SPrecDev to Dev (*p* = .025) in NT controls. In FCz, main effects of Age (*F*_(1,77)_ = 10.19, *p* = .002, *η*^2^ = .12), where amplitudes increased with age, and Group (*F*_(1,77)_ = 4.76, *p* = .032, *η*^2^ = .06) were observed, indicating that SPrecDev amplitude was higher in individuals with FXS (*p* = .009). For latency, main effects of Age were observed in Cz (*F*_(1,84)_ = 11.67, *p* < .001, *η*^2^ = .12) and FCz (*F*_(1,84)_ = 5.55, *p* = .021, *η*^2^ = .06), indicating shorter latencies with age.

#### MMN

In Cz, no effects were observed. A main effect of Group was observed in FCz (*F*_(1,84)_ = 6.66, *p* = .012, *η*^2^ = .07), and MMN amplitude was higher in individuals with FXS (*p* = .012). For latencies, main effects of Group were observed in Cz (*F*_(1,81)_ = 20.68, *p* < .001, *η*^2^ = .20) and FCz (*F*_(1,84)_ = 7.46, *p* = .008, *η*^2^ = .08), and latencies were longer in individuals with FXS in Cz (*p* < .001) and FCz (*p* = .008).

### Correlations Between AEPs and Clinical Symptoms in FXS

#### Cognitive Functioning (Leiter-3)

**Fig. 2:**
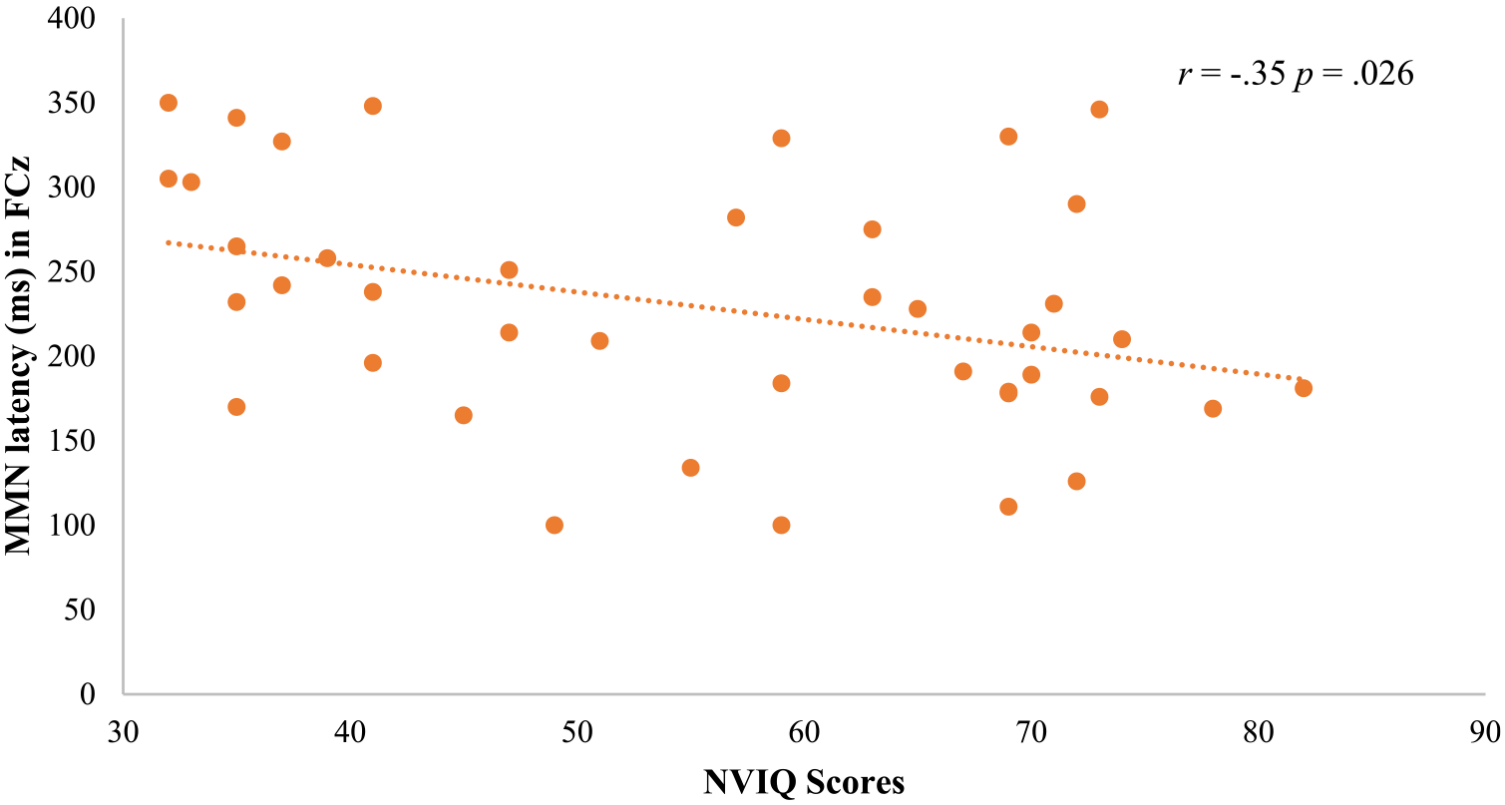
Scatterplot for NVIQ and MMN latency in FCz

No differences in NVIQ were found between males and females with FXS (*F*_(1,40)_ = 1.40, *p* = .24, *η*^2^ = .18). NVIQ correlated with P1 latency of Dev in Cz (*r* = − .33, *p* = .036), with N2 latency of SPrecDev in FCz (*r* = − .36, *p* = .022), and with MMN latency in FCz (*r* = − .35, *p* = .026; Fig. 2), indicating that shorter latencies are associated with better cognitive functioning.

#### Autistic Symptoms (ADOS Social Affect + Restricted and Repetitive Behavior, ABC-C-FX Stereotypy, and ADAMS Obsessive/Compulsive)

**Fig. 3:**
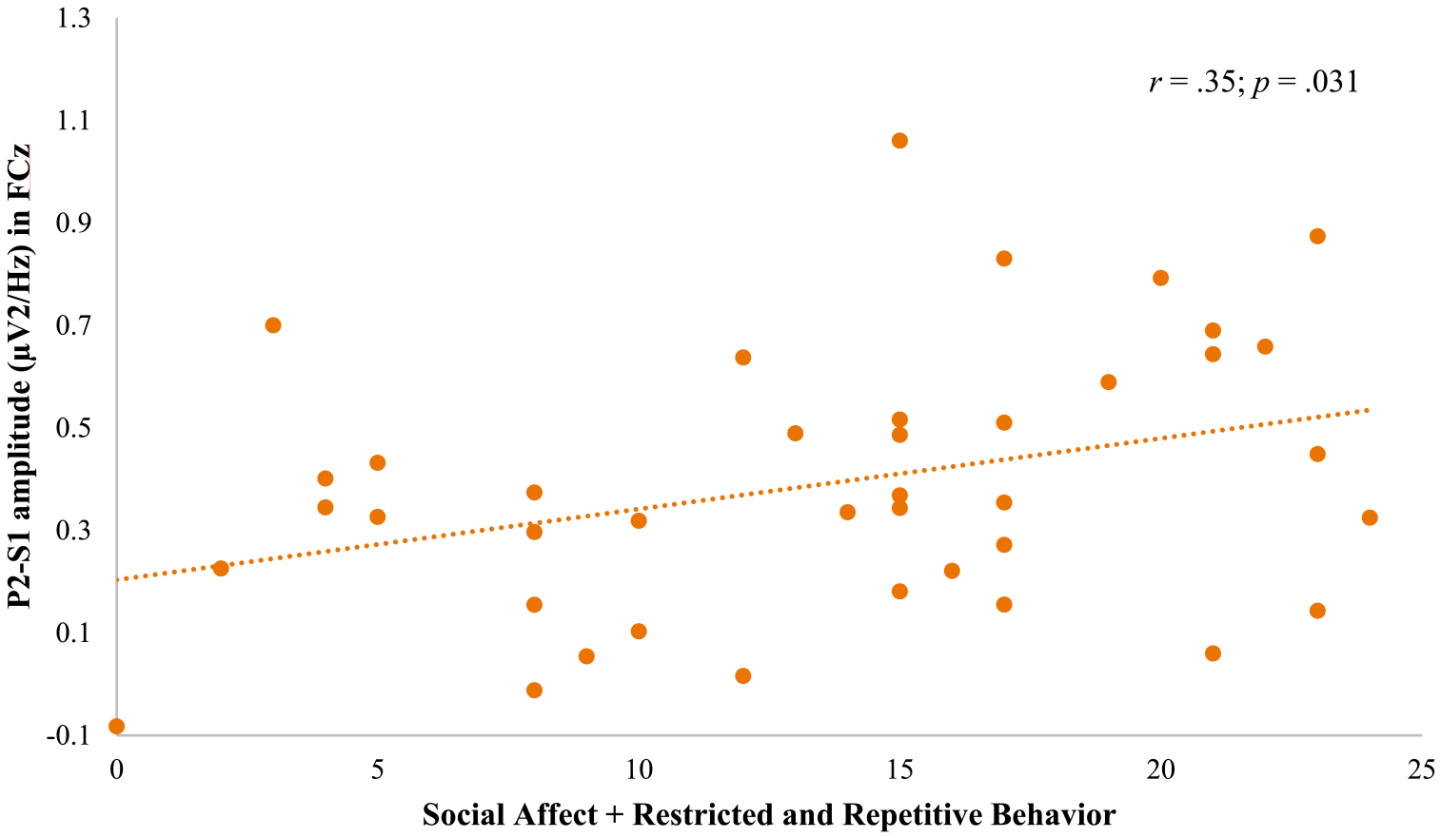
Scatterplot for social affect + restricted and repetitive behavior and P2-S1 in FCz

P2 peak amplitude of S1 correlated with Social Affect + Restricted and Repetitive Behavior in FCz (*r* = .35, *p* = .031; Fig. 3), with ABC-C-FX Stereotypy in FCz (*r* = .37, *p* = .031) and with ADAMS Obsessive/Compulsive in FCz (*r* = .35, *p* = .031). N2 peak amplitude of S1 correlated with ABC-C-FX Stereotypy in Cz (*r* = − .50, *p* = .003) and with ADAMS Obsessive/Compulsive in Cz (*r* = − .53, *p* = .003). P2 peak amplitude for SPrecDev correlated with Social Affect + Restricted and Repetitive Behavior in FCz (*r* = .42, *p* = .024), with ABC-C-FX Stereotypy in Cz (*r* = .37, *p* = .039) and FCz (*r* = .36, *p* = .029), and with ADAMS Obsessive/Compulsive in Cz (*r* = .45, *p* = .012) and FCz (*r* = .38, *p* = .028). P2 peak amplitude for Dev correlated with ABC-C-FX Stereotypy in Cz (*r* = .50, *p* = .003) and FCz (*r* = .45, *p* = .015). P3a peak amplitude of Dev correlated with ABC-CFX Stereotypy in Cz (*r* = .41, *p* = .036). Lastly, N2 latency of S1 correlated with ADAMS Obsessive/Compulsive in FCz (*r* = .42, *p* = .021). These results suggest that higher P2, N2 and P3a peak amplitudes, as well as a longer N2 latency, are associated with the presence of more severe autistic traits in our population.

#### Adaptive Behaviors (Vineland Daily Living Skills and Socialization Domains)

**Fig. 4:**
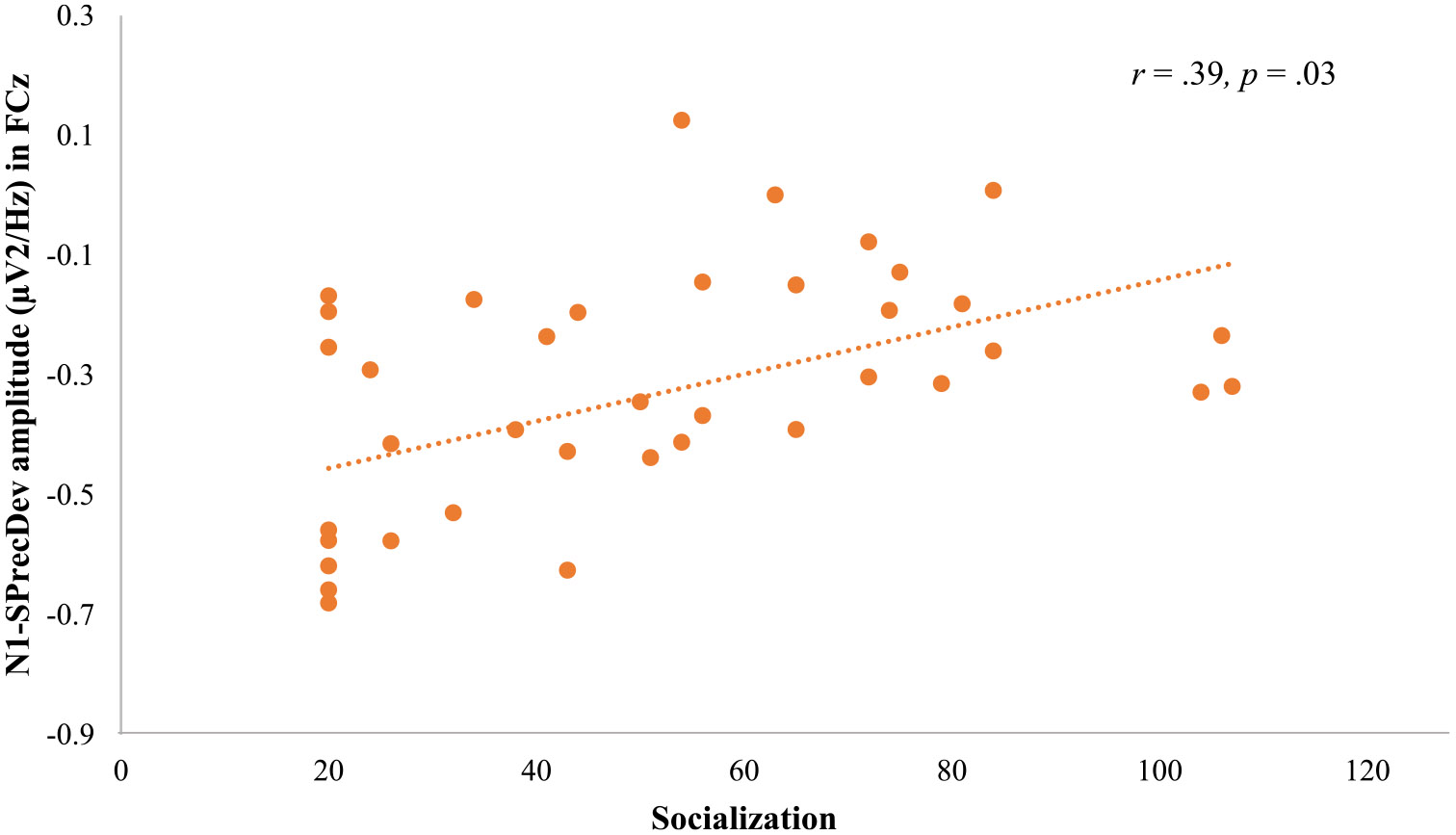
Scatterplot for socialization and N1-SPrecDev in FCz

N1 peak amplitude of SPrecDev correlated with Daily Living Skills in FCz (*r* = .35, *p* = .032), and with Socialization in Cz (*r* = .37, *p* = .046) and FCz (*r* = .39, *p* = .03; Fig. 4). P2 peak amplitude of SPrecDev correlated with Daily Living Skills in FCz (*r* = − .39, *p* = .016) and with Socialization in FCz (*r* = − .40, *p* = .016). Here, lower N1 and P2 peak amplitudes are associated with better adaptive behaviors.

#### Social Avoidance (ABC-C-FX and ADAMS Social Avoidance Subscales)

N2 amplitude of Dev correlated with ADAMS Social Avoidance in FCz (*r* = −.39, *p* = .034) and N2 latency of S1 correlated with ADAMS Social Avoidance in FCz (*r* = .38, *p* = .032), indicating that higher N2 amplitudes and longer N2 latencies are associated with more social deficits.

#### Anxiety and Depression (ADAMS General Anxiety and Depressed Mood Subscales)

MMN amplitude correlated with Depressed Mood in Cz (*r* = − .40, *p* = .028), suggesting that higher MMN peak amplitude is associated with more depressive symptoms. Lastly, N1 latency of Dev correlated with Depressed Mood in FCz (*r* = .40, *p* = .024), where a longer latency indicates more depressive symptoms.

#### ADHD Symptoms (ABC-C-FX Hyperactivity, ADAMS Manic/ Hyperactivity, and SNAP ADHD-Inattention and ADHD-Hyperactivity/Impulsivity)

P2 amplitude of S1 correlated withABC-C-FX Hyperactivity in Cz (*r* = .40, *p* = .025), with ADAMS Manic/Hyperactivity in Cz (*r* = .38, *p* = .025), and with SNAP ADHD-Hyperactivity/Impulsivity in Cz (*r* = .42, *p* = .025) and FCz (*r* = .43, *p* = .028). P2 amplitude of SPrecDev correlated with ABC-C-FX Hyperactivity in Cz (*r* = .40, *p* = .024), with the ADAMS Manic/Hyperactivity in Cz (*r* = .38, *p* = .024), with SNAP ADHD-Inattention in Cz (*r* = .35, *p* = .031), and with SNAP ADHD-Hyperactivity/Impulsivity in Cz (*r* = .47, *p* = .012). N2 amplitude of S1 correlated with the ADAMS Manic/ Hyperactivity in Cz (*r* = − .43, *p* = .014), and with SNAP ADHD-Inattention in Cz (*r* = − .37, *p* = .032), and with SNAP ADHD-Hyperactivity/Impulsivity in Cz (*r* = − .51, *p* = .004). These results suggest that higher P2 and N2 amplitudes are associated with more severe ADHD symptoms. N1 latency of S1 correlated with ABC-C-FX Hyperactivity in Cz (*r* = .56, *p* = .002), with ADAMS Manic/Hyperactivity in Cz (*r* = .53, *p* = .002), with SNAP ADHD-Inattention in Cz (*r* = .37, *p* = .019), and with SNAP ADHD-Hyperactivity/Impulsivity in Cz (*r* = .45, *p* = .005). N2 latency of S1 correlated with ABC-C-FX Hyperactivity in FCz (r = .38, *p* = .04), and with SNAP ADHD-Hyperactivity/Impulsivity in FCz (*r* = .40, *p* = .04). N1 latency of SPrecDev correlated with SNAP ADHD-Inattention in Cz (*r* = .40, *p* = .04), and with SNAP ADHD-Hyperactivity/Impulsivity in Cz (*r* = .37, *p* = .04). Lastly, N1 latency of Dev correlated with ABC-C-FX Hyperactivity in Cz (*r* = .44, *p* = .024). Here, longer N1 and N2 latencies indicate more severe ADHD symptoms in our population.

## Discussion

This study aimed to perform a comprehensive assessment of AEPs, habituation and change detection alterations in individuals with FXS with the main purpose of identifying how these atypicalities relate to various prominent clinical impairments frequently observed in this population. Firstly, it was observed that FXS participants showed higher amplitudes and longer latencies compared to NT controls for standard and deviant stimuli. As expected, habituation and change detection were observed in NT controls, whereas no such effects were found in individuals with FXS. Nevertheless, FXS participants presented a higher MMN peak amplitude compared to NT controls, but with longer latency. We also found several associations with the main clinical symptoms observed in FXS, namely cognitive functioning, autistic symptoms, adaptive behaviors, depression, as well as inattention, hyperactivity and aggressivity. Hence, our study contributed to a better understanding of the cortical auditory mechanisms associated with the main symptoms observed in individuals with FXS by establishing that many altered AEP components are associated with clinical anomalies in FXS.

### AEP Components

First, we confirmed AEP abnormalities in individuals with FXS. N1 and P2 AEP responses have been extensively investigated in FXS. The current study mainly replicated previous results by reporting exaggerated peak amplitudes for standard stimuli in individuals with FXS, as well as longer latencies for standard stimuli compared to NT controls (Ethridge et al., 2016, 2020; Knoth et al., 2014, 2018; Sinclair et al., 2017; Van der Molen et al., 2012a, 2012b). Results also revealed higher N1 and P2 peak amplitudes for deviant stimuli in FXS, which have been previously observed (Van der Molen et al., 2012a, 2012b), but no significant group differences in N2 amplitudes or latencies were found. Our results support the need for studies investigating N2 in individuals with FXS. Our study also provides new insights into P1 responses, the earliest sensory ERP component, of children and adults with FXS. Contrary to our hypotheses, individuals with FXS showed reduced P1 peak amplitudes and shorter latencies for standard stimuli compared to NT controls. So far, only two studies have explored P1 peak amplitude in FXS. One study found higher peak amplitudes in FXS children compared to NT children in the temporal region, but not in the frontal region (An et al., 2022), while another study investigating P1 responses in children and adults found no differences between individuals with FXS and NT controls in the fronto-central region (Ethridge et al., 2020). Here, it is possible that the large N1 peak amplitude occurring just after P1 is responsible for the decreased P1 peak amplitude in our FXS participants. Moreover, no study has ever reported P1 latency in FXS. As an early auditory processing component, the P1 should be systematically studied in AEP investigation as it could provide insights into early stages of sensory processing in FXS.

### Habituation and Change Detection Effects

In our study, no N1 habituation was found in either NT controls or FXS participants. However, the amplitudes of the P1 and P2 components showed habituation in NT controls but not in our FXS cohort. While we are the first to report P1 habituation alterations in FXS, P2 habituation results were inconsistent across previous studies (Côté et al., 2021; Ethridge et al., 2019), suggesting that distinct neural and cognitive mechanisms might account for different results regarding habituation processes in FXS. Firstly, FMRP is thought to interact with large conductance calcium- activated potassium (BK) channels in cortical excitatory neurons to regulate synaptic transmission (Deng et al., 2013). However, in FXS, lack of FMRP leads to reduced activation of BK channels, contributing to dendritic spine morphology abnormalities and altered short-term synaptic plasticity (Kshatri et al., 2020), which may impair habituation processes thus leading to learning (Typlt et al., 2013) and cognitive (Siegel et al., 2017) deficits. Further, as habituation to sensory stimuli implies a refractory period of neural mechanisms (Clearwater et al., 2008; Ethridge et al., 2016), it is possible that impaired synaptic plasticity contributes to deficient refractory periods in FXS. Taken together, these results suggest that disrupted habituation in individuals with FXS in the current study may be related to their relatively lower cognitive functioning (> 70% presenting with cooccurring ID; Table 2) when compared to previous studies that reported P2 habituation in FXS (Côté et al., 2021). Indeed, Knoth and colleagues (2018) identified that while delayed habituation occurred in FXS participants with moderate ID level, people with FXS who presented with more severe ID (NVIQ < 42), did not show habituation at all.

Consistent with our hypothesis, a change detection effect was found in NT controls, but not in individuals with FXS. So far, in FXS, very few studies have investigated the P3 component and contradictory results were obtained in these studies (St. Clair et al., 1987; Van der Molen et al., 2012a, 2012b). The nature of the tasks (passive vs active) and the distinction between the P3a and P3b components, which are thought to reflect different processes (Riby et al., 2008), might explain the inconsistent results. In the current study, NT controls showed a P3a change detection effect, and exaggerated P3a amplitudes were found in individuals with FXS for standard stimuli, replicating previous results (Van der Molen et al., 2012a, 2012b).

### The MMN

The MMN is thought to reflect cognitive processing and has been predominantly investigated in the auditory modality in FXS (Helfrich & Knight, 2019). In the passive oddball paradigm, MMN refers to the ability to distinguish between frequent and novel stimuli, and it involves a short-term memory trace (Luck, 2014). Only two other studies have investigated the MMN component in FXS so far, and the results are conflicting which may be attributed to different reasons. Firstly, the literature concerning the time at which MMN peaks is not well defined, and time-windows used in different studies vary widely. As we had hypothesized that individuals with FXS would have longer MMN latencies compared to NT controls, our time-window allowed to detect MMN peaks occurring later in our FXS participants. Moreover, it has been established that the MMN component overlaps with the N1 and N2 components (Näätänen et al., 2014). Here, N1 amplitudes were found to be higher in individuals with FXS compared to NT for both standard and deviant stimuli, and N2 amplitudes tended to be higher for the deviant stimulus. Thus, it is possible that larger negative peak amplitudes were generated in the calculation of the MMN component in our FXS population. Furthermore, Van der Molen and colleagues (2012a, 2012b) calculated the difference curve by subtracting the responses to all the standard stimuli from the responses to the deviant stimuli. However, in the current study, we used the same method as Ethridge and colleagues (2020), and only used the standard stimuli directly preceding the deviant tone. Lastly, it has been reported that in different brain pathologies, exaggerated MMN could be associated with excitability of the central nervous system (CNS) or the central auditory system (Näätänen et al., 2014). Hence, our results of increased MMN in individuals with FXS are in line with the extensive evidence of cortical hyperexcitability and auditory hypersensitivity in both animal models and humans with FXS (Ethridge et al., 2017; Garcia-Pino et al., 2017; Hagerman et al., 2012; Rotschafer & Razak, 2014; Sinclair et al., 2017).

### Associations Between Auditory Processing EEG Markers and Clinical Symptoms in FXS

Considering the high prevalence of ID in our FXS cohort, investigating the effects of NVIQ on AEP components was imperative. Notably, even the female participants with FXS in our sample demonstrated comparatively low cognitive functioning due to the inclusion criteria of the clinical trial. Thus, no difference in NVIQ was found between male and female FXS participants. We first observed that a longer P1 latency for the deviant stimulus was linked to a lower NVIQ in our participants with FXS. Literature on the P1 component is far from extensive, but one study observed a longer P1 latency in individuals with Williams Syndrome (WS), which, just like FXS, is characterized by cooccurring ID (Fagundes Silva et al., 2021). Another study found increased P1 latencies in individuals with ASD, as well as negative correlations between P1 latency and cognitive abilities (Russo et al., 2009). Furthermore, it has been reported that P1 latencies tend to decrease with age, suggesting that longer latencies might be related to delayed brain maturation (Sinclair et al., 2017). As P1 latency was shorter in FXS for standard tones in the current study, but increased P1 latency for deviant tones was found to be related to lower NVIQ in FXS, our results imply that P1 latency and its relation to cognitive deficits in FXS might be specifically tied to the discrimination process between frequent and infrequent tones. N2 latency for the stimuli preceding the deviant tones was also found to be longer in participant manifesting lower NVIQ. Longer N2 latencies have previously been reported in individuals WS (Nascimento et al., 2024) and Down syndrome (DS) (César et al., 2010), two genetic disorders characterized by ID, suggesting that prolonged N2 latencies might be related to altered cognitive functioning. We also observed that a prolonged MMN latency was associated with a lower NVIQ. While previous results regarding MMN latency in ID are contradicting, there is evidence of longer MMN latency in individuals with ID (Ikeda et al., 2000). A more recent study also observed a longer MMN latency in individuals with DS who manifested poorer cognitive abilities (Saini et al., 2023). Combined with our initial finding of longer MMN latencies in central and fronto-central regions in individuals with FXS compared to NT controls, the association found between longer MMN latency and lower NVIQ suggests that MMN latency might be a potential dose–response marker of cognitive impairment in FXS.

Next, we investigated how the severity of autistic symptoms correlated with impaired auditory processing in our cohort. We observed that higher P2 amplitudes for both standard and deviant stimuli were strongly associated with more severe autistic traits. Our results are consistent with a recent study reporting enhanced P2 amplitudes in adults with ASD (Borgolte et al., 2021). P2 amplitudes are associated with cortical hyperexcitability and hypersensitivity to auditory stimuli, which are thought to be caused by deficient GABAergic signaling (Bear et al., 2004; Rubenstein & Merzenich, 2003). As these characteristics are present in both FXS and ASD (Devitt et al., 2015), it is possible that the exaggerated P2 peaks found in FXS are even more prevalent in individuals with FXS manifesting severe ASD symptoms. Exaggerated N2 and P3a amplitudes and longer N2 latencies were also found to be related to the severity of autistic symptoms in our FXS population. These AEP components are thought to reflect attentional and inhibitory control and have been reported to be increased in children with ASD (Faja et al., 2016; Ferri et al., 2003; Høyland et al., 2017). Whether our results are the manifestation of attentional and inhibitory control difficulties often found in ASD or to other subphenotype of ASD would need further investigation.

As mentioned previously, exaggerated N1 and P2 amplitudes are robust markers of altered AEPs in FXS. Here, N1 and P2 amplitudes for standard stimuli were higher in individuals with FXS compared to NT controls, and both markers were related to lower adaptive functioning. These results could suggest that individuals with FXS who present lower N1 and P2 responses, might exhibit enhanced adaptive functioning, including personal and social skills, due to decreased cortical excitability. Furthermore, as no correlations were found between N1and P2 peak amplitudes of the first standard stimuli and adaptive behaviors, it implies that it is really the reduction in responses to repeated stimuli—the habituation, that is associated with improved adaptive behaviors. This result bridges the gap between habituation as a neuronal learning phenomenon and adaptive abilities as they are presented in the phenotype.

Anxiety and social withdrawal are core features in FXS (Cordeiro et al., 2011), while depressive symptoms are also fairly common (Chonchaiya et al., 2009). Here, elevated MMN amplitude was associated with higher scores on the Depressed Mood subscale in the central region. Altered MMN has been consistently reported in patients suffering from bipolar disorders (BD) and depression (Chen et al., 2015; He et al., 2010; Kähkönen et al., 2007; Kaur et al., 2011; Kim et al., 2020; Qiao et al., 2013; Restuccia et al., 2016; Takei et al., 2009). Given that MMN studies are scarce and conflicting in FXS, our results warrant the need for future research taking the presence of depressive symptoms into consideration when investigating the MMN component in individuals with FXS. Moreover, our results showed that prolonged N1 latency for the deviant stimulus was associated with more depressive symptoms, which is consistent with a recent study reporting longer N1 latencies in adolescents with MDD (Wen et al., 2021). Lastly, an increase in N2 peak amplitude and a longer N2 latency was associated with social avoidance. Enhanced N2 amplitudes have been reported in children manifesting behavioral inhibition (Lahat et al., 2014), while a prolonged N2 latency was associated with antisocial behaviors (Delfin et al., 2022). Taken together, these results suggest that alterations of the N2 component might be a correlate of impaired social behaviors.

Given that more than 60% of individuals with FXS present ADHD symptoms (Cregenzán-Royo et al., 2022), we assessed core symptoms of ADHD in our FXS population. Firstly, we observed that P2 and N2 peak amplitudes were higher in individuals with FXS presenting more severe inattentive and hyperactive symptoms. Findings regarding the P2 and N2 components in ADHD are inconsistent in the literature, but studies have found increased P2 amplitudes during a visual task (Zarka et al., 2021) and during an auditory oddball paradigm (Oades et al., 1996), while one study reported enhanced P2 amplitudes in adults with ADHD during an active visual task (Prox et al., 2007). As P2 and N2 peak amplitudes are thought to reflect inhibitory processes, our results suggest altered inhibition in individuals with FXS presenting ADHD symptoms when processing sensory information. Our correlational analyses also showed that longer N1 and N2 latencies were associated with severe ADHD symptoms, which is in line with the longer N1 and N2 latencies reported in children with ADHD Combined (Johnstone et al., 2001). Furthermore, these authors reported an increase in N1 latencies for standard stimuli with age in the ADHD Inattentive subtype, and generally longer N1 latencies in the ADHD Combined subtype across all ages, while NT children showed a decrease in N1 latencies, indicating atypical maturation of N1 latency in ADHD. These results suggest altered attentional processes in individuals with ADHD. Longer N1 latencies have been seldom observed in individuals with FXS, but we found a longer latency for the first standard stimuli in our FXS cohort in the current study. Overall, our results indicate that individuals with FXS presenting more severe symptoms of ADHD might also manifest impaired attentional mechanisms implicated in the detection of auditory stimuli.

### Strengths and Limitations

Obtaining the data of 41 participants having performed EEG is quite a significant sample given the rarity of FXS in the general population. One major limitation of this study is the heterogeneity of our sample, where both males and females with FXS, and a wide age range were included. However, the similarities in cognitive and behavioral symptoms between our male and female participants with FXS constitutes a strength of this study, since impairments and sex effects are less entangled. Furthermore, the inclusion of such a broad age range is a common occurrence in the FXS literature. Supplementary File 5 provides a table with the age and sex distributions in prior studies investigating ERPs in individuals with FXS, as well as age and sex effects reported in these studies. While the current study established important associations between AEPs and clinical symptoms in FXS, the impact of age and sex will need to be further investigated in order to better unravel these effects on auditory processing.

## Conclusions

This study confirms that several AEP components are altered in a population of children and adults with FXS. Specifically, N1 and P2 amplitudes and latencies were found to be significantly higher in FXS, and no habituation or change detection effects were observed in this population. Interestingly, we observed that N1 and P2 alterations were associated with the presence of symptoms predominantly related to the FXS phenotype, namely autistic and ADHD symptoms. AEP alterations in general might thus reflect phenotype severity, with more extreme AEP alterations predicting increased symptomatology. Lastly, we observed an association between lower cognitive abilities and longer MMN latencies, suggesting that MMN latency could be a potential biomarker of impaired cognitive functioning in FXS.

## Supplementary Material

supplementaryfile_5

supplementaryfile_3

supplementaryfile_4

supplementaryfile_1

supplementaryfile_2

Supplementary Information The online version contains supplementary material available at https://doi.org/10.1007/s10803-025-07076-4.

## Figures and Tables

**Table 1:** Demographics of the study population

	FXS	Controls
N	41	46
Males (n, %)	31 (75.61%)	21 (45.65%)
Females (n, %)	10 (24.39%)	25 (54.35%)
Age		
Mean ± SD	16.95 ± 7.75	15.00 ± 9.69
Range	7–34	5–30

**Table 2:** Cooccurring diagnoses in the clinical population

Cooccurring diagnoses	N
ID	
None: ≥ 70 (%)	10 (24.39%)
Mild: 50–69 (%)	14 (34.15%)
Moderate: 36–49 (%)	10 (24.39%)
Severe: 20–35 (%)	7 (17.07%)
Profound: < 20 (%)	0 (0%)
ASD	
Non-autism (%)	10 (24.39%)
Autism (%)	31 (75.71%)
ADHD	
No ADHD (%)	24 (58.54%)
ADHD (%)	17 (41.46%)

**Table 3:** Pharmacological treatments in the clinical population

Pharmacological treatments	
Antidepressants	
N (%)	12 (29.27%)
Psychostimulants	
N (%)	16 (39.02%)
