# Supplementary material for: Associations Between Altered Auditory EEG Markers and Clinical Impairments in Fragile X Syndrome
Source: J Autism Dev Disord. Author manuscript; Available in PMC 2026 Mar 27. (PMC13022936; doi:10.1007/s10803-025-07076-4)
Supplement: supplementaryfile_5 [file NIHMS2150840-supplement-supplementaryfile_5.docx]

**Supplementary file 5.** Samples, age and sex effects in prior studies.

| Study | FXS | | NT Ctrls | | Age effects | | Sex effects |
| --- | --- | --- | --- | --- | --- | --- | --- |
|  | **N**  **(M:F)** | **Age in years**  **(mean±SD)** | **N**  **(M:F)** | **Age in years**  **(mean±SD)** |  |  | |
| St. Clair et al., 1987 |  |  |  |  |  |  | |
|  | 28  (26:2) | 16-66 (43**±**13) | 83 (unknown) | 18-75  (unknown) | No correlations with age | NA | |
| Castrèn et al., 2003 |  |  |  |  |  |  | |
|  | 5  (5:0) | 7-21  (unknown) | 4  (4:0) | Unknown  (10.06**±**0.6) | NA | NA | |
| Van der Molen et al., 2012 | 16  (16:0) | 18-42  (29.6) | 20  (20:0) | 19-47  (29.2) | NA | NA | |
| Van der Molen et al., 2012^2^ | 16  (16:0) | 18-42  (29.6) | 22  (22:0) | 19-47  (29.2) | NA | NA | |
| Knoth et al., 2014 |  |  |  |  |  |  | |
|  | 12  (8:4) | 10-22  (14.7±13.75) | 12  (9:3)  9  (6:3) | 11-32  (16.09±6.02)  5-7  (5.8±0.83) | NA | Higher NVIQ in females with FXS  No differences in AEP components | |
| Knoth et al., 2018 |  |  |  |  |  |  | |
|  | 14  (10:4) | 9-32  (15.5±6.06) | 26  (15:11) | 9-32  (17.1±6.1) | Age was not a significant predictor in the models | NA | |
|  |  |  |  |  |  |  | |
| Ethridge et al., 2016 | 14  (11:3) | 14-57  (28.5±11.7) | 15  (10:5) | 16-55  (28.9±10.2) | NA | Sex was not a significant predictor in the model | |
| Ethridge et al., 2020 |  |  |  |  |  |  | |
|  | 41  (28:13) | 4-51  (17.3±8.9) | 27  (16:11) | 4-54  (21±10.4) | Similar trajectories for FXS and NT Ctrls for all components except P2 amplitude | Females with FXS modulate P2 latency more similarly to NT females | |
| Côté et al., 2021 | 14  (9:5) | 3-30  16.43±6.41) | 55  (20:35) | 3-30  (14.51±7.71) | 4 age groups were formed:  1) 3-4YO  2) 5-10YO  3) 11-17YO  4) 18-30YO  No differences were found in AEP components between the groups | No sex effects were found | |
