# Supplementary material for: Associations Between Altered Auditory EEG Markers and Clinical Impairments in Fragile X Syndrome
Source: J Autism Dev Disord. Author manuscript; Available in PMC 2026 Mar 27. (PMC13022936; doi:10.1007/s10803-025-07076-4)
Supplement: supplementaryfile_3 [file NIHMS2150840-supplement-supplementaryfile_3.docx]

**Supplementary file 3. Summary of AEP results between FXS and NT Ctrls.**

| AEP | Interactions | Effects | Pairwise comparisons | Age correlations |
| --- | --- | --- | --- | --- |
| P1 standard stimuli |  |  |  |  |
| Amplitudes | In Cz :  Habituation*Group interaction (*F*_(1,79)_ = 5.02, *p* = .028, *η^2^* = .06) | In Cz: Age effect (*F*_(1,79)_ = 6.12, *p* = .015, *η^2^* = .07)  In FCz: Age effect (*F*_(1,78)_ = 4.78, *p* = .032, *η^2^* = .06) | In Cz: S1 lower in FXS (*p* = .01), habituation in NT Ctrls (*p* = .014)  In FCz: Habituation in NT Ctrls (*p* = .036) | S1 in NT Ctrls in Cz (*r* = -.34, *p* = .022) and FCz (*r* = -.33, *p* = .028)  SPrecDev in NT Ctrls in Cz (*r* = -.37, *p* = .012) |
| Latencies | NS | In Cz: Group effect *F*_(1,84)_ = 11.38, *p* = .001, *η^2^* = .12)  In FCz: Group effect *F*_(1,84)_ = 4.98, *p* = .028, *η^2^* = .06) | In Cz: Shorter latencies in FXS for both stimulus types (S1: *p* = .013; SPrecDev: *p* = .008) | S1 in FXS in FCz (*r* = .39, *p* = .013) |
| N1 standard stimuli |  |  |  |  |
| Amplitudes | NS | In Cz: Age (*F*_(1,80)_ = 31.71, *p* < .001, *η^2^* = .28) and Group (*F*_(1,80)_ = 38.25, *p* < .001, *η^2^* = .32) effects  In FCz: Age (*F*_(1,81)_ = 35.25, *p* < .001, *η^2^* = .30) and Group (*F*_(1,81)_ = 33.63, *p* < .001, *η^2^* = .29) effects | Higher amplitudes in FXS for both stimulus types in both ROIs (all *p* < .001) | SPrecDev in FXS in Cz (*r* = -.34, *p* = .029) and FCz (*r* = -.34, *p* = .034)  S1 in NT Ctrls in Cz (*r* = -.60, *p* < .001) and FCz (*r* = -.68, *p* < .001)  SPrecDev in NT Ctrls in Cz (*r* = -.58, *p* < .001) and FCz (*r* = -.56, *p* < .001) |
| Latencies | In FCz :  Latency*Group interaction (*F*_(1,83)_ = 7.00, *p* = .01, *η^2^* = .08) | In Cz: Group effect (*F*_(1,84)_ = 13.85, *p* < .001, *η^2^* = .14)  In FCz: Group effect (*F*_(1,83)_ = 7.12, *p* = .009, *η^2^* = .08) | In Cz: Longer SI latency in FXS (*p* < .001)  In FCz: Longer SI latency in FXS (*p* < .001) | S1 in FXS in FCz (*r* = -.35, *p* = .028)  S1 in NT Ctrls in FCz (*r* = .34, *p* = .02) |
| P2 standard stimuli |  |  |  |  |
| Amplitudes | NS | In Cz: Group effect (*F*_(1,84)_ = 6.86, *p* = .01, *η^2^* = .08)  In FCz: Age (*F*_(1,79)_ = 4.17, *p* = .044, *η^2^* = .05) and Group (*F*_(1,79)_ = 13.66, *p* < .001, *η^2^* = .15) effects | In Cz: Both stimulus types higher in FXS (S1: *p* = .032; SPrecDev: *p* = .014)  In FCz: Both stimulus types higher in FXS (S1: *p* = .015; SPrecDev: *p* < .001). Small habituation in NT Ctrls (*p* = .05) | SPrecDev in FXS in FCz (*r* = .35, *p* = .035) |
| Latencies | NS | In Cz: Group effect *F*_(1,83)_ = 16.77, *p* < .001, *η^2^* = .17)  In FCz: Group effect (*F*_(1,84)_ = 22.52, *p* < .001, *η^2^* = .21) | Longer S1 latencies in FXS in both ROIs (*p* < .001) and longer SPrecDev latencies in Cz (*p* = .015) and FCz (*p* = .008) | S1 in NT Ctrls in FCz (*r* = .41, *p* = .005)  SPrecDev in NT Ctrls in Cz (*r* = .36, *p* = .015) and FCz (*r* = .39, *p* = .008) |
| N2 standard stimuli |  |  |  |  |
| Amplitudes | NS | In Cz: Age effect (*F*_(1,81)_ = 7.91, *p* = .006, *η^2^* = .09)  In FCz: Age effect (*F*_(1,80)_ = 16.67, *p* < .001, *η^2^* = .17) | NS | S1 in NT Ctrls in Cz (*r* = .38, *p* = .01) and FCz (*r* = .48, *p* < .001)  SPrecDev in NT Ctrls in Cz (*r* = .37, *p* = .013) and FCz (*r* = .47, *p* = .001) |
| Latencies | NS | In Cz: Age effect (*F*_(1,84)_ = 10.71, *p* = .002, *η^2^* = .11)  In FCz: Age (*F*_(1,84)_ = 9.86, *p* = .002, *η^2^* = .11) and Group (*F*_(1,84)_ = 4.51, *p* = .037, *η^2^* = .05) effects | NS | S1 in NT Ctrls in Cz (*r* = .40, *p* = .007)  SPrecDev in FXS in FCz (*r* = .40, *p* = .01) |
| P1 deviant stimulus |  |  |  |  |
| Amplitudes | NS | In Cz: Age effect ((*F*_(1,81)_ = 7.18, *p* = .009, *η^2^* = .08)  In FCz: Age effect (*F*_(1,80)_ = 5.90, *p* = .017, *η^2^* = .07) | NS | In NT Ctrls in FCz (*r* = -.30, *p* = .046) |
|  |  |  |  |  |
| Latencies | NS | In Cz: Group effect *F*_(1,84)_ = 5.56, *p* = .021, *η^2^* = .06) | NS | In FXS in Cz (*r* = .33, *p* = .036)  In NT Ctrls in Cz (*r* = -.31, *p* = .035) |
| N1 deviant stimulus |  |  |  |  |
| Amplitudes | NS | In Cz: Age (*F*_(1,81)_ = 19.51, *p* < .001, *η^2^* = .19) and Group (*F*_(1,81)_ = 27.81, *p* < .001, *η^2^* = .26) effects | In Cz: Higher Dev amplitude in FXS (*p* < .001) | In NT Ctrls in Cz (*r* = -.48, *p* = .001) and FCz (*r* = -.59, *p* < .001) |
|  |  | In FCz: Age (*F*_(1,81)_ = 35.83, *p* < .001, *η^2^* = .31) and Group *F*_(1,81)_ = 15.55, *p* < .001, *η^2^* = .16) effects | In FCz: Higher Dev amplitude in FXS (*p* = .01) |  |
| Latencies | NS | NS | NS | In NT Ctrls in Cz (*r* = .32, *p* = .033) |
| P2 deviant stimulus  Amplitudes | NS | In Cz: Group effect (*F*_(1,81)_ = 7.85, *p* = .006, *η^2^* = .09)  In FCz: Group effect (*F*_(1,76)_ = 19.19, *p* < .001, *η^2^* = .20) | In Cz: Higher Dev amplitude in FXS (*p* = .02)  In FCz: Higher Dev amplitude in FXS (*p* = .002) | NS |
| Latencies | NS | In Cz: Age effect (*F*_(1,84)_ = 7.66, *p* = .007, *η^2^* = .08)  In FCz: Age (*F*_(1,84)_ = 11.81, *p* < .001, *η^2^* = .12) ) and Group *F*_(1,84)_ = 10.26, *p* = .002, *η^2^* = .11) effects | NS | In NT Ctrls in Cz (*r* = .40, *p* = .001) and FCz (*r* = .59, *p* < .001) |
| N2 deviant stimulus |  |  |  |  |
| Amplitudes | NS | In Cz: Age (*F*_(1,81)_ = 6.06, *p* = .016, *η^2^* = .07) and Group (*F*_(1,81)_ = 5.20, *p* = .025, *η^2^* = .025) effects  In FCz: Age effect (*F*_(1,79)_ = 6.94, *p* = .01, *η^2^* = .08) | NS | In NT Ctrls in FCz (*r* = .31, *p* = .038) |
| Latencies | NS | In Cz: Age effect (*F*_(1,84)_ = 5.42, *p* = .022, *η^2^* = .06)  In FCz: Age (*F*_(1,84)_ = 5.00, *p* = .028, *η^2^* = .06) and Group (*F*_(1,84)_ = 5.52, *p* = .021, *η^2^* = .06) effects | NS | NS |
| P3 stimuli  Amplitudes | NS | In Cz: Age effect (*F*_(1,73)_ = 5.83, *p* = .018, *η^2^* = .07)  In FCz: Age *F*_(1,77)_ = 10.19, *p* = .002, *η^2^* = .12 and Group (*F*_(1,77)_ = 4.76, *p* = .032, *η^2^* = .06) effects | In Cz:  Change detection in NT Ctrls (*p* = .025)  In FCz: Higher SPrecDev amplitudes in FXS (*p* = .009) | Dev in NT Ctrls in Cz (*r* = .44, *p* = .003) and FCz (*r* = .41, *p* = .005) |
| Latencies | NS | In Cz: Age effect (*F*_(1,84)_ = 11.67, *p* < .001, *η^2^* = .12)  In FCz: Age effect (*F*_(1,84)_ =5.55, *p* = .021, *η^2^* = .06) | NS | SPrecDev in NT Ctrls in FCz (*r* = -.30, *p* = .046)  Dev in NT Ctrls in Cz (*r* = -.45, *p* = .002) and FCz (*r* = -.39, *p* = .007) |
| MMN  Amplitudes | NA | In FCz: Group effect (*F*_(1,84)_ = 6.66, *p* = .012, *η^2^* = .07) | In FCz: Higher in FXS (*p* = .012) | NS |
| Latencies | NA | In Cz: Group effect (*F*_(1,81)_ = 20.68, *p* < .001, *η^2^* = .20)  In FCz: Group effect (*F*_(1,84)_ = 7.46, *p* = .008, *η^2^* = .08) | Latencies higher in FXS in Cz (*p* < .001) and FCz (*p* = .008) | NS |
