# Supplementary material for: Associations Between Altered Auditory EEG Markers and Clinical Impairments in Fragile X Syndrome
Source: J Autism Dev Disord. Author manuscript; Available in PMC 2026 Mar 27. (PMC13022936; doi:10.1007/s10803-025-07076-4)
Supplement: supplementaryfile_4 [file NIHMS2150840-supplement-supplementaryfile_4.docx]

**Supplementary file 4. Correlations between AEP components and clinical phenotype in FXS.**

| Phenotypic manifestation | Correlations |
| --- | --- |
| Cognitive functioning |  |
|  | P1 latency of Dev in Cz (*r* = -.33, *p* = .036),  N2 latency of SPrecDev in FCz (*r* = -.36, *p* = .022)  MMN latency in FCz (*r* = -.35, *p* = .026) |
| Autistic symptoms |  |
|  | P2 peak amplitude of S1 with Social Affect + Restricted and Repetitive Behavior in FCz (*r* = .35, *p* = .031), with ABC-C-FX Stereotypy in FCz (*r* = .37, *p* = .031), and with ADAMS Obsessive/Compulsive in FCz (*r* = .35, *p* = .031)  P2 peak amplitude of SPrecDev with Social Affect + Restricted and Repetitive Behavior in FCz (*r* = .42, *p* = .024), with ABC-C-FX Stereotypy in Cz (*r* = .37, *p* = .039) and FCz (*r* = .36, *p* = .029), and with ADAMS Obsessive/Compulsive in Cz (*r* = .45, *p* = .012) and FCz (*r* = .38, *p* = .028)  P2 peak amplitude of Dev with ABC-C-FX Stereotypy in Cz (*r* = .50, *p* = .003) and FCz (*r* = .45, *p* = .015)  N2 peak amplitude of S1 with ABC-C-FX Stereotypy in Cz (*r* = -.50, *p* = .003) and with ADAMS Obsessive/Compulsive in Cz (*r* = -.53, *p* = .003)  P3 peak amplitude of Dev with ABC-CFX Stereotypy in Cz (*r* = .41, *p* = .036)  N2 latency of S1 with ADAMS Obsessive/Compulsive in FCz (*r* = .42, *p* = .021) |
| Adaptive behaviors |  |
|  | N1 peak amplitude of SPrecDev with Daily Living Skills in FCz (*r* = .35, *p* = .032), and with Socialization in Cz (*r* = .37, *p* = .046) and FCz (*r* = .39, *p* = .03)  P2 peak amplitude of SPrecDev with Daily Living Skills in FCz (*r* = -.39, *p* = .016) and with Socialization in FCz (*r* = -.40, *p* = .016) |
| Social avoidance |  |
|  | N2 amplitude of Dev with ADAMS Social Avoidance in FCz (*r* = -.39, *p* = .034)  N2 latency of S1 with ADAMS Social Avoidance in FCz (*r* = .38, *p* = .032) |
| Anxiety and depression |  |
|  | MMN amplitude with Depressed Mood in Cz (*r* = -.40, *p* = .028)  N1 latency of Dev with Depressed Mood in FCz (*r* = .40, *p* = .024) |
| ADHD symptoms |  |
|  | P2 amplitude of S1 with ABC-C-FX Hyperactivity in Cz (*r* = .40, *p* = .025), with ADAMS Manic/Hyperactivity in Cz (*r* = .38, *p* = .025), and with SNAP ADHD-Hyperactivity/Impulsivity in Cz (*r* = .42, *p* = .025) and FCz (*r* = .43, *p* = .028).  P2 amplitude of SPrecDev with ABC-C-FX Hyperactivity in Cz (*r* = .40, *p* = .024), with the ADAMS Manic/Hyperactivity in Cz (*r* = .38, *p* = .024), with SNAP ADHD-Inattention in Cz (*r* = .35, *p* = .031), and with SNAP ADHD-Hyperactivity/Impulsivity in Cz (*r* = .47, *p* = .012)  N2 amplitude of S1 correlated with the ADAMS Manic/Hyperactivity in Cz (*r* = -.43, *p* = .014), and with SNAP ADHD-Inattention in Cz (*r* = -.37, *p* = .032), and with SNAP ADHD-Hyperactivity/Impulsivity in Cz (*r* = -.51, *p* = .004)  N1 latency of S1 with ABC-C-FX Hyperactivity in Cz (*r* = .56, *p* = .002), with ADAMS Manic/Hyperactivity in Cz (*r* = .53, *p* = .002), with SNAP ADHD-Inattention in Cz (*r* = .37, *p* = .019), and with SNAP ADHD-Hyperactivity/Impulsivity in Cz (*r* = .45, *p* = .005)  N1 latency of SPrecDev correlated with SNAP ADHD-Inattention in Cz (*r* = .40, *p* = .04), and with SNAP ADHD-Hyperactivity/Impulsivity in Cz (*r* = .37, *p* = .04)  N1 latency of Dev with ABC-C-FX Hyperactivity in Cz (*r* = .44, *p* = .024)  N2 latency of S1 correlated with ABC-C-FX Hyperactivity in FCz (r = .38, *p* = .04), and with SNAP ADHD-Hyperactivity/Impulsivity in FCz (*r* = .40, *p* = .04) |
