# Supplementary material for: Associations Between Altered Auditory EEG Markers and Clinical Impairments in Fragile X Syndrome
Source: J Autism Dev Disord. Author manuscript; Available in PMC 2026 Mar 27. (PMC13022936; doi:10.1007/s10803-025-07076-4)
Supplement: supplementaryfile_1 [file NIHMS2150840-supplement-supplementaryfile_1.docx]

**Supplementary file 1. Neuropsychological instruments’ scores in the clinical population.**

| Instrument | Scores |  |
| --- | --- | --- |
| Leiter-III |  | |
| Mean±SD | 55.37±15.55 | |
| Range | 32-82 | |
| ADOS-2 |  | |
| Social Affect + Restricted and Repetitive Behavior (Mean±SD) | 13.49±6.54 | |
| VABS-3 |  | |
| Daily Living Skills (Mean±SD) | 55.82±28.00 | |
| Socialization (Mean±SD) | 51.03±26.54 | |
| ABC-C-FX |  | |
| Stereotypy (Mean±SD) | 5.00±4.76 | |
| Hyperactivity (Mean±SD) | 9.27±7.37 | |
| Social Avoidance (Mean±SD) | 3.22±2.60 | |
| ADAMS |  | |
| Manic/Hyperactive (Mean±SD) | 6.32±4.10 | |
| Depressed Mood (Mean±SD) | 3.17±3.35 | |
| Social Avoidance (Mean±SD) | 8.17±4.33 | |
| General Anxiety (Mean±SD) | 6.78±3.55 | |
| Obsessive/Compulsive (Mean±SD) | 2.49±2.44 | |
| SNAP-IV |  | |
| ADHD Inattention (Mean±SD) | 15.10±6.05 | |
| ADHD Hyperactivity/Impulsivity (Mean±SD) | 9.15±6.72 | |
