# Supplementary material for: Associations Between Altered Auditory EEG Markers and Clinical Impairments in Fragile X Syndrome
Source: J Autism Dev Disord. Author manuscript; Available in PMC 2026 Mar 27. (PMC13022936; doi:10.1007/s10803-025-07076-4)
Supplement: supplementaryfile_2 [file NIHMS2150840-supplement-supplementaryfile_2.docx]

**Supplementary file 2. Percentage of kept trials between the groups.**

|  | FXS | Controls |
| --- | --- | --- |
| S1 |  |  |
| % of kept trials (mean±SD) | 67.28±17.55 | 68.40±18.99 |
| ANOVA | *F*(1,85) = .081, *p =* .78 | |
| SPrecDev |  |  |
| % of kept trials (mean±SD) | 65.06±15.19 | 65.22±17.93 |
| AVOVA | *F*(1,85) = .002, *p =* .97 | |
| Dev  % of kept trials (mean±SD) | 65.35±16.84 | 66.71±19.68 |
| ANOVA | *F*(1,85) = .119, *p* = .73 | |
